# Downregulation of miR-22 Contributes to Epithelial-Mesenchymal Transition in Osteosarcoma by Targeting Twist1

**DOI:** 10.3389/fonc.2020.00406

**Published:** 2020-04-24

**Authors:** Shu-tao Zhu, Xiao Wang, Jun-yi Wang, Guang-hui Xi, Yang Liu

**Affiliations:** Department of Orthopedics, Huaihe Hospital, The First Affiliated Hospital of Henan University, Kaifeng, China

**Keywords:** miR-22, EMT, metastasis, twist1, osteosarcoma

## Abstract

The epithelial-mesenchymal transition (EMT) is a vital step in osteosarcoma (OS) progression toward metastasis, but the specific molecular events governing this process are incompletely characterized, with miRNAs having increasingly been found to regulate the EMT. In this study, We assessed levels of miR-22 and its target, Twist1, via real-time PCR (qRT-PCR). We further used functional proliferation assays, measures of cell morphology, and western blotting to assess the functional relevance of miR-22 in OS and confirmed Twist1 as a miR-22 target via luciferase reporter assay. We observed a significant decrease in miR-22 levels in OS tumor samples relative to normal tissue, with such downregulating being significantly associated with tumor histological grade. When overexpressed, miR-22 impaired OS cell proliferation and EMT progression. We found Twist1 to be a direct miR-22 target, with levels of miR-22 and Twist1 mRNA being inversely correlated in patient samples. When overexpressed, miR-22 suppressed Twist1 translation and thereby attenuated the EMT in OS cells. These results clearly demonstrate that miR-22 can regulate the EMT in OS cells via targeting Twist1, thus highlighting a potentially novel pathway that can be therapeutically targeted in order to treat OS.

## Introduction

Osteosarcoma (OS) is a highly prevalent form of bone cancer that most frequently impacts adolescents and younger children (1), in whom it is the second most prominent cancer-associated cause of death (2). Recent advances in surgical and chemo/radiotherapy have improved OS patient 5-year survival to 70% (3). The remaining 30% of patients, however, often exhibit high rates of local tumor recurrence, early development of tumor metastases, and limited tumor chemosensitivity, ultimately contributing to the poor outcomes in these individuals (4). There is therefore a clear need to explore the molecular mechanisms governing OS so as to highlight potential novel biomarkers of tumor development or progression, and to thereby develop better means of treating this deadly disease.

MicroRNAs (miRNAs) are small RNA molecules which lack coding potential, yet which are able to readily mediate the post-translational regulation of target mRNA expression via suppressing translation or inducing degradation (5). These miRNAs are capable of regulating a broad array of cellular processes, such as differentiation, apoptotic death, or proliferation with specific miRNAs serving to suppress or promote tumor function in a context-dependent manner (6–8). Epithelial-to-mesenchymal transition (EMT) is an essential process tightly associated with the tumor metastatic progression in a range of tumor types (9). This EMT process is associated with a reduction in the epithelial-like features of cancer cells and the acquisition of mesenchymal-like features that are necessary to mediate effective invasion and migration. ZEB1, Snail, and Twist1 are three key transcription factors known to regulate the EMT, with all three acting to repress the transcription of E-cadherin. Certain miRNAs have been shown to be capable of regulating the EMT, with miR-200 having been shown to be capable of repressing drug resistance and an epithelial phenotype in tumor cells (10, 11). Such evidence indicates that specific miRNAs are capable of modulating the EMT. Whether such miRNAs are able to modulate the EMT in the context of OS, however, remains incompletely understood, and as such further research into the role of miRNAs in this setting is vital as it has the potential to highlight novel therapeutic interventions for OS.

Several tumor types have been found to exhibit miR-22 dysregulation. For example, Zhang et al. determined that in breast cancer, miR-22 is able to target Sirt1 and thereby suppress oncongenesis and enhance cancer cell radiosensitivity (12). Xin et al. found miR-22 to target ATP citrate lyase and to thereby inhibit the growth and metastasis of OS, prostate cancer, lung cancer, and cervical cancer cells (13). The role of miR-22 in the progression of OS and in the EMT process, however, has yet to be assessed.

In the present study we explored the mechanistic role of miR-22 in OS. We determined that OS patient tissues exhibited significantly lower miR-22 levels than did normal paired tissue, and that this expression was negatively correlated with tumor stage. We further found that when overexpressed, miR-22 disrupted OS cell proliferation and the EMT via targeting the EMT-associated transcription factor Twist1. These results offer new insight into the molecular mechanisms whereby OS develops, and suggest that targeting this miR-22/Twist1 pathway may have therapeutic utility.

## Materials and Methods

### Human Samples

Pairs of human OS tissue samples and matched normal tissue controls were obtained from the Department of Orthopedics, Huaihe Hospital, the First Affiliated Hospital of Henan University. All samples were collected surgically, and were snap frozen with liquid nitrogen prior to analysis. We classified the samples according to Enneking system which is the common staging systems for malignant bone tumors (Table 1). The Institutional Review Board and Ethics Committee of Henan University approved this study, with patients having given written consent to participate.

**Table 1:** Clinical Information of Patients.

**Characteristic**	**Histological grade**		
	**Stage I**	**Stage II**	**Stage III-IV**
Total Patient No.	11	11	10
**Age (years)**			
>25	3	5	4
≤25	8	6	6
**Gender**			
Male	9	10	8
Female	2	1	2
Location			
Proximal	7	8	4
Distal	4	3	6
**Tumor size**			
≤5 cm	8	4	4
>5 cm	3	7	6
Metastasis	0	0	10

### Cell Culture and Transduction

The HOS and MG63 OS cell lines from ATCC were grown in RPMI-1640, whereas HEK-293T cells were grown in DMEM containing 10% FBS and penicillin/streptomycin at 37°C in a 5% CO2 incubator.

A lentiviral packaging kit (Open Biosystems, AL, USA) was used to produce lentiviruses bearing either miR-22 or negative control (NC) based on provided directions. HEK-293T cells were used for packaging. Supernatants were collected 48 h after co-transfection of these cells, and lentiviral supernatants were combined for 24 h with OS cell lines together with polybrene (2.5 μg/ml). Following transduction, stably transduced cells were selected using puromycin (1.5 μg/ml).

### qRT-PCR

Trizol (Invitrogen, CA, USA) was used to isolate sample RNA according to provided direction. The stem-loop-specific primer approach was used for assessing miR-22 expression, with U6 used for normalization. Twist1 expression was assessed via using the RT Reagent Kit (Vazyme, China) to reverse transcribe total RNA, and GAPDH was used for normalizing gene expression. SYBR Green Master Mix (Vazyme, China) was used for all qRT-PCR reactions, with relative quantification (2^−ΔΔ*Ct*^) used for all fold change calculations.

### Western Blotting

RIPA buffer containing protease inhibitors was employed to lyse cells, with supernatant protein levels being assessed via BCA assay (Beyotime, Jiangsu, China). For tissue samples, tissue was ground in liquid nitrogen before RIPA lysis, followed by processing as above. Protein samples were then separated via SDS-PAGE, transferred to a PVDF membrane (Roche, Switzerland), and subjected to Western blotting based on provided directions. The ECL Detection System (Thermo Scientific, IL, USA) was used for signal detection. Antibodies against E-cadherin and Twist1 were from Cell Signaling Technology (MA, USA). Anti-GAPDH was from Bioworld Technology (GA, USA), and anti-Vimentin was from Abcam (Cambridge, UK).

### Luciferase Reporter Assay

Cells were plated in triplicate wells of 24-well plates for 24 h, after which the appropriate plasmids were transfected into the cells along with the pRL-TK Renilla plasmid using Lipofectamine 2,000 (Life Technologies). Following a 24 h incubation, the Dual-Luciferase Reporter Assay (Promega) was conducted based on provided directions.

### Statistics

GraphPad Prism 5 was used for all analyses, with experiments independently performed three times. T-tests were used to compare all results, with *P* < 0.05 as the significance threshold. The Pearson's rank test was used to assess the relationship between miR-22 and Twist1 in human OS tissue samples.

## Results

### OS Tumors Exhibit Reduced miR-22 Expression Correlated With More Advanced Disease

**Figure 1:**
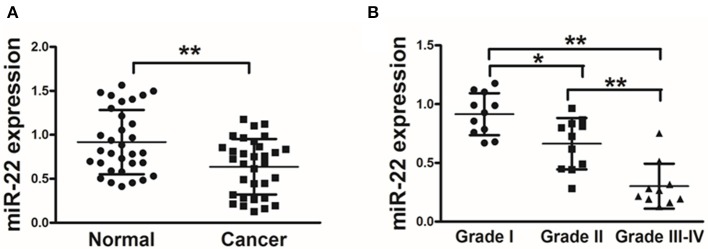
OS patient samples exhibit reduced miR-22 expression associated with more advanced disease. **(A)** qRT-PCR was used to assess miR-22 expression relative to U6 (for normalization) in 60 OS tissue pairs. **(B)** Relative miR-22 expression as a function of cancer stage. Data are means±SD of 3 replicates. *P < 0.05; **P < 0.01.

We first assessed miR-22 expression in 32 paired human OS and normal tissue control samples via stem-loop qRT-PCR. We found that OS tissues exhibited a marked reduction in miR-22 expression relative to adjacent normal control samples (Figure 1A). We further found that there was a negative correlation between miR-22 expression and tumor histological grade (Figure 1B). This suggests that lower expression of miR-22 corresponds to a more advanced stage of OS.

### miR-22 Suppresses the Proliferation and EMT of OS Cells

**Figure 2:**
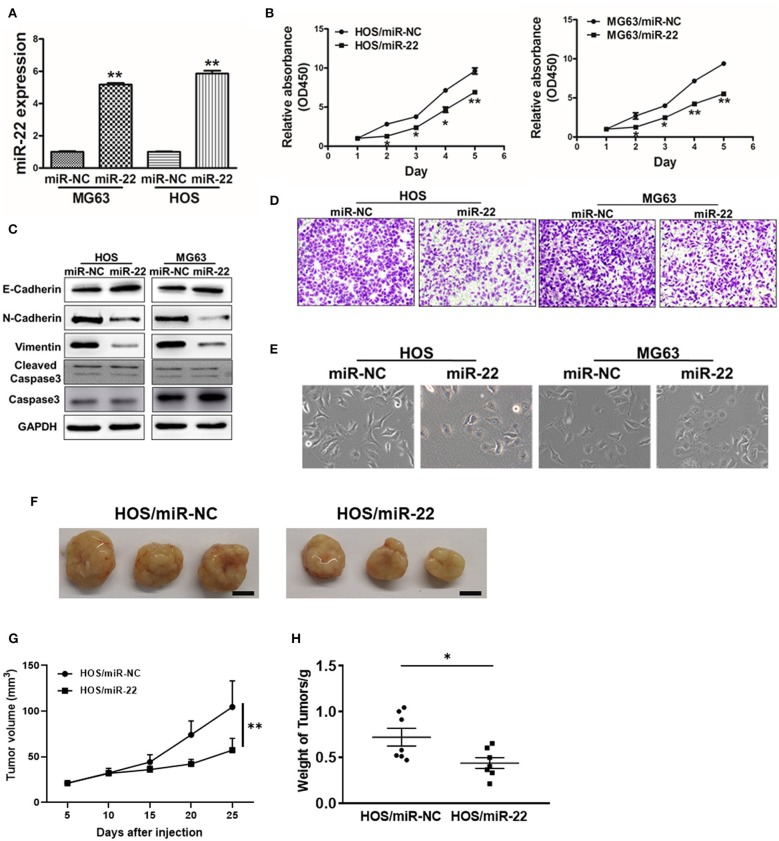
miR-22 suppresses proliferation and EMT in OS cells. **(A)** MG63 and HOS OS cell lines stably expressing miR-22 were assessed via qRT-PCR to confirm miR-22 expression. **(B)** A CCK8 assay was used to assess the proliferation of the indicated OS cells. **(C)** Western blotting was used to assess E-cadherin, N-cadherin, Vimentin, Caspase 3 and Cleaved caspase 3 levels in these cells. **(D)** Chambers of transwells covered with Matrigel were used for Invasion assays. **(E)** MG63 and HOS cells were assessed via phase contrast microscopy, with those overexpressing miR-22 exhibiting a shift from a spindle-like to a round/cobblestone morphology. **(F–H)** Female BALB/c nude mice were subcutaneously injected with 10^6^ HOS cells harboring miR-NC or miR-22 overexpression. Tumor volume and weight were monitored over time as indicated, and the tumor was excised and weighed after 25 days. Bar = 10 mm. Data are means±SD of 3 replicates. *P < 0.05; **P < 0.01.

We next assessed the effects of miR-22 on OS cell proliferation and metastasis via generating human OS cell lines (HOS and MG63) stably expressing miR-22 or negative control (Figure 2A). We found that miR-22 overexpression significantly reduced cell proliferation relative to NC controls not due to the effect on apoptosis (Figures 2B,C).

We further observed significant morphological changes in MG63 and HOS cells overexpressing miR-22, with a shift from a spindle-shaped morphology to a rounder/cobblestone appearance (Figure 2E). We then measured the EMT markers vimentin, N-cadherin and E-cadherin via western blotting, revealing them to be significantly decreased and increased, respectively, in OS cells overexpressing miR-22. Meanwhile, the invasion ability of OS cells expressing miR-22 is weaker to the control cells (Figure 2D). We also performed the *in vivo* assays, the results showed that miR-22 will indeed reduce cell proliferation abilities (Figures 2F,G). These results therefore suggested that miR-22 is capable of suppressing the proliferation and EMT of OS cells.

### miR-22 Targets Twist1

**Figure 3:**
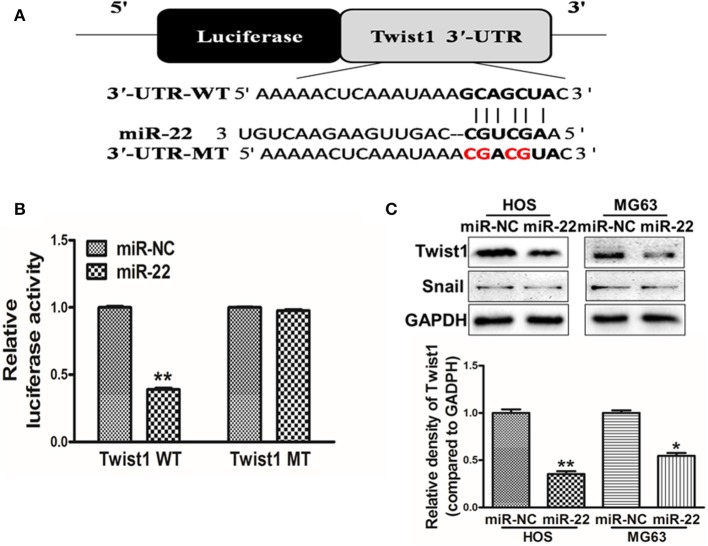
Twist1 is a miR-22 target. **(A)** The putative site of miR-22 binding within the Twist1 3'-UTR is show, with WT and mutant (MT; in red) constructs for these 3'-UTR regions being generated and cloned into a pMIR luciferase reporter vector. **(B)** The pMIR-Twist1-WT and pMIR-Twist1-MT vectors were transfected into HOS cells along with miR-22 or control, and after 48 h luciferase activity was assessed. **(C)** Twist1 protein levels in MG63/HOS cells were assessed via Western blotting. Data are means±SD of 3 replicates. *P < 0.05; **P < 0.01.

To further explore the mechanisms whereby miR-22 regulates OS cell activity, we utilized the Targetscan tool to identify possible miR-22 target genes. One such predicted target was Twist1 (Figure 3A), which is a key transcription factor associated with the EMT and with metastasis. To confirm the ability of miR-22 to target Twist1, we generated luciferase reporter plasmids containing a WT or mutated version of the Twist1 3'-UTR containing the putative miR-22 binding site. We found that miR-22 was able to significantly reduce the luciferase activity of the WT but not mutated plasmid construct, consistent with its binding to the target region (Figure 3B). Western blotting further revealed that overexpressing miR-22 decreased Twist1 protein levels, but not snail levels which has been reported the miR-22-target in other cancer cell lines (Figure 3C). Together these findings show that miR-22 can specifically interact with the 3'-UTR of Twist1 to regulate its expression in OS cells.

### Twist1 and miR-22 Are Negatively Correlated in Human OS

**Figure 4:**
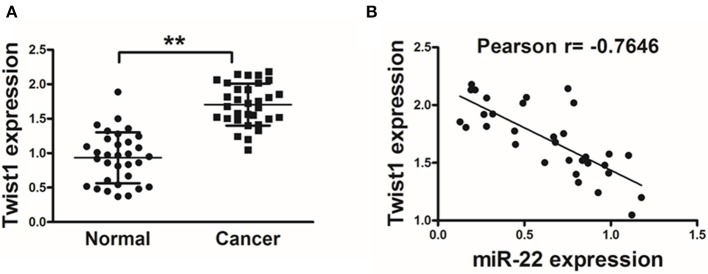
Twist1 expression negatively correlated with that of miR-22 in human OS. **(A)** qRT-PCR was used to assess Twist1 expression in human OS samples, with GAPDH used for normalization. **(B)** The association between Twist1 and miR-22 expression in OS samples was assessed via Pearson′s correlation analysis. Data are means±SD of 3 replicates. **P < 0.01.

In order to establish the clinical relevance of these results, we next explored Twist1 expression in human OS tissue samples, revealing a significant increase in Twist1 expression in tumor tissues relative to paired normal control tissues (Figure 4A). A Pearson's correlation analysis between Twist1 and miR-22 levels in OS samples further revealed that there was a strong negative correlation between the expression of these two factors (Pearson's correlation *r* = −0.7646) (Figure 4B).

### miR-22 Regulates OS Cell Proliferation and EMT via Targeting Twist1

**Figure 5:**
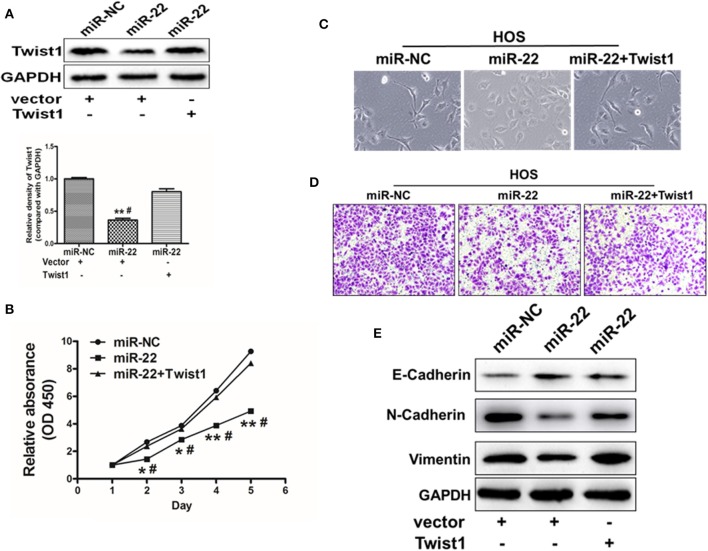
miR-22 targets Twist1 to regulate the EMT in OS cells. **(A)** Twist1 overexpression in HOS cells was partially able to reverse the effects of overexpressing miR-22. **(B)** A CCK8 assay was used to assess OS cell proliferation, revealing that Twist1 overexpression in HOS cells partially overcame the inhibition of proliferation induced upon miR-22 overexpression. **(C)** Images of changes in cell morphology in cells miR-22 mimics or control with our without Twist1 overexpression. **(D)** Chambers of transwell covered by Matrigel were used for invasion assays. **(E)** Western blotting was used to measure E-cadherin N-cadherin and Vimentin expression in HOS cells. Data are means ± SD of 3 replicates. *P < 0.05; **P < 0.01 vs. miR-NC control. ^#^*P* < 0.05 vs. miR-22 + Twist.

We finally sought to explore whether there was a direct functional link between miR-22 and Twist1 in the context of the regulation of OS cell proliferation and EMT. To test this, we co-transfected HOS cells using both miR-22 or NC along with a pCMV-Twist1 construct for 48 h. Overexpression of Twist1 lacking of the 3'-UTR miR-22 binding site was able to partially reverse the effects of miR-22 overexpression, with increased Twist1 expression (Figure 5A), enhanced proliferation (Figure 5B), increased invasion abilities (Figure 5D) and a restoration of normal cellular morphology (Figure 5C) and N-cadherin, vimentin protein levels upon Twist1 overexpression (Figure 5E). These results thus indicate that miR-22 is capable of targeting Twist1 in order to regulate the proliferation and EMT of OS cells.

## Discussion

For past several decades, the survival of osteosarcoma patients has been mostly improved. Despite evidence of genomic instability and a high frequency of chromothripsis and kataegis, osteosarcomas carry few recurrent therapeutic gene targets. Meanwhile, the developments of targeted agents have also been generally disappointing (14). Thus, the underlying mechanism remain largely unknow. Several cancers have been found to exhibit miRNA dysregulation, and such altered miRNA activity has in turn been found to play a functional role in tumor progression, with particular miRNAs promoting or suppressing cancer progression. In the present report we found that human OS patient samples exhibited significantly reduced miR-22 expression relative to normal tissue controls, with decreased expression corresponding to increasing tumor histological grade. We further found that overexpressing miR-22 led to a significant repression of the EMT in OS cells, with Twist1 being a direct miR-22 target in these cells, thereby modulating EMT progression. This is the first study we are aware of demonstrating the ability of miR-22 to regulate Twist1 in OS, although further study of this signaling pathway is needed to validate these results.

The EMT is a key process in driving cancer metastasis (15), with several transcription factors including Twist1, Snail, and ZEB1 cooperating to control this complex process (15). The EMT is a common event that is observed in a wide range of cancer types undergoing metastasis (16). Epithelial-to-Mesenchymal Transition (EMT) is a dynamic, invertible and relative process. Many studies have indicated that, in tumor cells, “Epithelial, E” and “Mesenchymal, M” are not at the completely different poles. Oppositely, in most case, tumor cells can reside in an intermediate state termed “metastable” phenotype between the epithelial and mesenchymal stages enabling them to undergo EMT- or MET related processes under specific conditions, which likely contributes to their aggressive clinical behavior. Although osteosarcoma cells arise from cells that descend from the mesenchyme. The osteosarcoma cells still maintain partial epithelial characters, like some epithelial markers, which are necessary to mediate cohesiveness during migration, and allows resistance to the mechanical stress that cells experience during migration (17, 18). Although it remains largely unknown that how osteosarcoma cells with a metastable phenotype can shift EMT- and MET-related processes, several studies have shown first clues. For instance, in osteosarcoma cells, the EMT-TFs, such as Twist, Snails, and Zebs are over-activated (19) and the genes such as TIM3 (20), ST6GAL1 (21), TRIM66 (22), UHRF1 (23), and CYR61 (24) contribute to the positive regulation of EMT-TFs. Moreover, miRNAs are implicated in the suppression and induction of EMT-related processes in osteosarcoma (25, 26). For instance, miR-370 and miR-132 suppress EMT-related processes inhibiting tumor growth and metastasis via targeting fork head box protein M1 (FOXM1) and SRY-box 4 (SOX4), respectively (27, 28). In the present report we observed reduced Vimentin expression and increased E-cadherin expression in cells overexpressing miR-22, with this overexpression corresponding to a significant reduction in OS cell EMT capacity. We further found that miR-22 directly targeted Twist1 to mediate this repression of the EMT, and this was further confirmed to be a clinically relevant result as Twist1 expression was negatively correlated with that of miR-22 in patient samples. We have thus shown that miR-22 expression is closely linked with the regulation of the EMT process in OS cells.

Twist1 is a helix-loop-helix transcription factor that plays essential roles in osteoblasts, myoblasts, and mesodermal cells, regulating their differentiation nad migration activity (29–32). Twist1 further promotes tumor cell proliferation, invasion, and resistance to cell death, in addition to enhancing EMT progression and angiogenic development (33–35). Twist1 upregulation is observed in a variety of cancer types, including OS as well as breast, bladder, gastric, and ovarian cancer (36–40).

## Conclusion

In conclusion, our results reveal that miR-22 is downregulated in human osteosarcoma in a manner that correlates with enhanced tumor progression an metastasis. We found that miR-22 was able to directly suppress its target Twist1, thereby impairing the proliferation and EMT of OS cells. These insights highlight the potential of this miR-22/Twist1 signaling axis to be used either as a prognostic biomarker or therapeutic target in OS.

## Data Availability Statement

All datasets generated for this study are included in the article/supplementary material.

## Ethics Statement

The studies involving human participants were reviewed and approved by The Institutional Review Board and Ethics Committee of Henan University. The patients/participants provided their written informed consent to participate in this study.

## Author Contributions

YL and SZ designed the study, analyzed data, and drafted the manuscript. XW, JW, and GX participated in the manuscript preparation and carried out the experiments *in vitro* and *in vivo*. All authors read and approved the final manuscript.

## Conflict of Interest

The authors declare that the research was conducted in the absence of any commercial or financial relationships that could be construed as a potential conflict of interest.
